# Performance of Health Workers in the Management of Seriously Sick Children at a Kenyan Tertiary Hospital: Before and after a Training Intervention

**DOI:** 10.1371/journal.pone.0039964

**Published:** 2012-07-31

**Authors:** Grace W. Irimu, David Gathara, Dejan Zurovac, Harrison Kihara, Christopher Maina, Julius Mwangi, Dorothy Mbori-Ngacha, Jim Todd, Alexandra Greene, Mike English

**Affiliations:** 1 Department of Paediatrics and Child Health, College of Health Sciences, University of Nairobi, Nairobi, Kenya; 2 Centre for Geographic Medicine Research – Coast, KEMRI/Wellcome Trust Research Programme, Kilifi, Kenya; 3 Centre for Tropical Medicine, Nuffield Department of Clinical Medicine, University of Oxford, Oxford, United Kingdom; 4 Center for Global Health and Development, Boston University, Boston, Massachusetts, United States of America; 5 Kenyatta National Hospital, Nairobi, Kenya; 6 London School of Hygiene and Tropical Medicine, London, United Kingdom; 7 Child Health, University of Dundee, Dundee, United Kingdom; 8 Department of Paediatrics, University of Oxford, Oxford, United Kingdom; University of Massachusetts Medical School, United States of America

## Abstract

**Background:**

Implementation of WHO case management guidelines for serious common childhood illnesses remains a challenge in hospitals in low-income countries. The impact of locally adapted clinical practice guidelines (CPGs) on the quality-of-care of patients in tertiary hospitals has rarely been evaluated.

**Methods and Findings:**

We conducted, in Kenyatta National Hospital, an uncontrolled before and after study with an attempt to explore intervention dose-effect relationships, as CPGs were disseminated and training was progressively implemented. The emergency triage, assessment and treatment plus admission care (ETAT+) training and locally adapted CPGs targeted common, serious childhood illnesses. We compared performance in the pre-intervention (2005) and post-intervention periods (2009) using quality indicators for three diseases: pneumonia, dehydration and severe malnutrition. The indicators spanned four domains in the continuum of care namely assessment, classification, treatment, and follow-up care in the initial 48 hours of admission. In the pre-intervention period patients' care was largely inconsistent with the guidelines, with nine of the 15 key indicators having performance of below 10%. The intervention produced a marked improvement in guideline adherence with an absolute effect size of over 20% observed in seven of the 15 key indicators; three of which had an effect size of over 50%. However, for all the five indicators that required sustained team effort performance continued to be poor, at less than 10%, in the post-intervention period. Data from the five-year period (2005–09) suggest some dose dependency though the adoption rate of the best-practices varied across diseases and over time.

**Conclusion:**

Active dissemination of locally adapted clinical guidelines for common serious childhood illnesses can achieve a significant impact on documented clinical practices, particularly for tasks that rely on competence of individual clinicians. However, more attention must be given to broader implementation strategies that also target institutional and organisational aspects of service delivery to further enhance quality-of-care.

## Introduction

Despite availability of World Health Organization (WHO) case management guidelines, studies in low-income settings continue to identify poor health workers' compliance with evidence-based standards and poor follow-up care as some of the problems facing paediatric service delivery [1], [2]. Perhaps linked to this, mortality rates in some of the hospitals are as high as 15% [2], [3], [4], [5]. Many of these deaths could be averted by giving timely, appropriate and safe hospital care to the seriously sick child [2], [6], [7].

In an effort to reduce childhood mortality in hospitals in Kenya, the Ministry of Health in collaboration with stakeholders developed ‘*Basic Paediatric Protocols*’ consisting of clinical practice guidelines (CPGs). The guidelines aim to improve paediatric emergency and admission care in the initial 48 hours of hospitalisation [8], [9]. The CPGs were derived and adapted from international and local disease specific guidelines [10], [11]. Subsequently an in-service training programme called “Emergency Triage Assessment and Treatment Plus admission care” (ETAT+), was developed to facilitate implementation of the CPGs. A detailed description of ETAT+ is provided elsewhere [8], [9].

Though the CPGs and ETAT+ were initially intended for district hospitals, they were in demand also at higher level hospitals [12]. Introduction of CPGs and ETAT+ training to KNH initially occurred in response to demand. This was followed by a more deliberate approach to disseminate the CPGs and provide ETAT+ training in partnership with Kenyatta National Hospital (KNH) and University of Nairobi (UoN), but with limited resources. In this study we evaluated effects of their introduction on clinical practices in a university teaching hospital. We address this question by quantitative examination of health workers' clinical performance in accordance with indicators agreed upon by the hospital staff as important markers of correct care.

## Methods

### Study design

This was an uncontrolled before and after design that included an attempt to explore any intervention dose-effect relationship. We applied this design because of the absence of comparable tertiary hospitals in Kenya.

### Study site

The study was conducted in KNH, a tertiary level hospital and a teaching hospital for the University of Nairobi (UoN) Medical School and other healthcare training institutions. KNH is the second largest hospital in Africa with a bed capacity of 1,800. The general paediatric wards have total capacity of 240 beds although the bed occupancy is often over 100%. There are 14,000 paediatric admissions annually. Sixty to seventy five trainee paediatricians who are enrolled in a three year postgraduate paediatric training programme in the UoN, provide most clinical in-patient care. They are normally supervised by 25 paediatricians. There are 126 qualified nurses who provide care on the four general paediatric wards – twelve to twenty nurses per shift. After initial emergency clerkship and initiation of life-saving treatments at the Paediatric Emergency Unit, all seriously sick patients are admitted to the wards and reviewed by a trainee paediatrician who provides the initial management plan.

### Selection of the target diseases and study intervention

**Figure 1:**
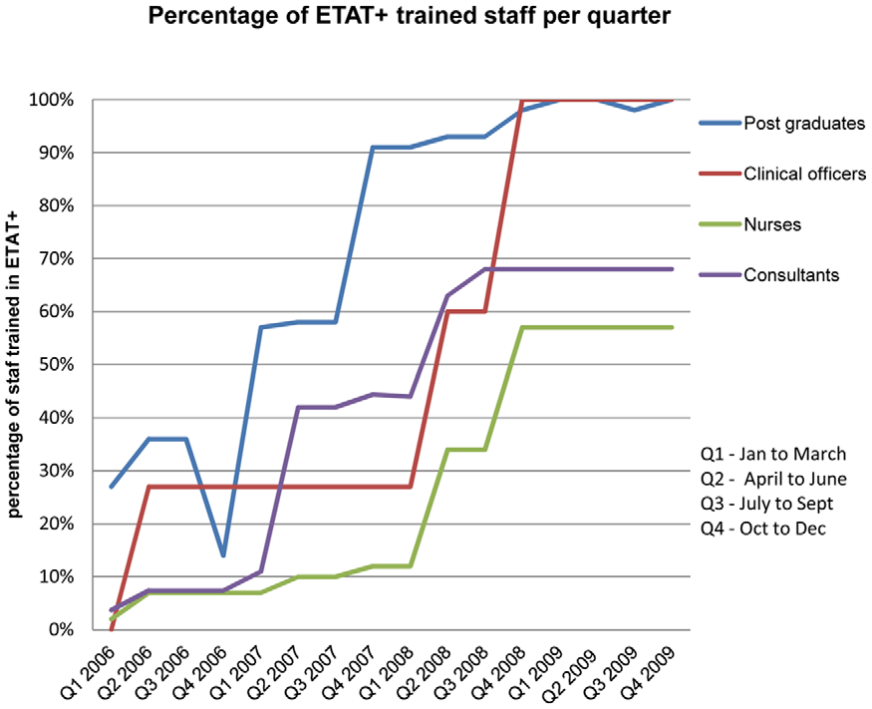
Coverage of 5 day ETAT+ training among the front-line service providers in the paediatrics wards and Paediatric Emergency Unit (PEU).

We focussed on medical records of patients with any of the three discharge diagnoses: pneumonia, dehydration (due to diarrhoea) and severe malnutrition. These three diseases have a high disease burden, high case fatality and explicit WHO/Kenyan guidelines for their case management that have largely remained unchanged since 2000. The primary intervention consisted of provision and wide dissemination of CPG booklets linked to a 5 day ETAT+ training targeting clinical and nursing care providers. CPG booklets and training were provided at no charge to KNH. ETAT+ training was escalated in response to demand rather than as part of an overarching strategy. In this before and after intervention study, we define a pre-intervention period as from January to December 2005 – before any introduction of CPGs or ETAT+ training; and a post-intervention period as from January to December 2009 when all junior clinicians, 70% of consultants, and 60% of nurses were already trained in ETAT+ (Figure 1). To explore the pattern of change, we divided the entire study duration (2005–09) into four interrelated periods defined by the intensity of intervention. Period 0 (2005) is the pre-intervention period and period 1 (2006) is piloting of the intervention. Period 2 (Jan 2007 to June 2008) is scaling up dissemination of the CPGs/training without active promotion of uptake of the best-practice recommendations. Period 3 (July 2008 –Dec 2009) comprised continued scaling up of dissemination of CPGs/training combined with efforts to reinforce adherence to best-practice recommendations. These activities included audit and feedback (17 sessions) and educational sessions (37 sessions) over the 18 months comprising period 3. These were part of additional participatory action research that will be reported in detail elsewhere. In brief, the educational sessions focussed on problems identified in the audit. Audit meetings were not necessarily focused on or tailored to the 15 key indicators reported here but were part of efforts to improve care for acute admissions. The sessions were cadre specific and targeted professionals such as nurses, nutritionists and trainee paediatricians who participated in the care of the seriously ill child.

### Sample size and sampling

We aimed to study 280 randomly selected medical records per year per disease (70 records per disease per quarter). This would detect an increase in correct practice for each disease from 50% in the pre-intervention period to 65% in the post-intervention period, significant at the 5% level with 90% power (with no adjustment for multiple comparisons). We used a modified multistage random sampling of eligible medical records to select episodes of illness treatment for the three target diseases. It is possible that a re-admitted child or one diagnosed with both pneumonia and dehydration was randomly included in more than one diagnostic group but only one diagnostic episode was assessed per admission. Patients with co-morbidity or complications that rendered treatment in accordance with CPGs inappropriate were excluded. Details of the process of selection of the study population including a full description of the inclusion and exclusion criteria are provided elsewhere (http://idoc-africa.org/documents/download/id/209).

### Data collection

Data were collected from free text medical records for the period January 2005– December 2009 using a direct electronic data entry tool and detailed standard operating procedures. Data entry used a web scripting language (Hypertext Pre-processor, PHP) with a Structured Query Language database (MySQL) backend (http://idoc-africa.org/documents/download/id/209). All the data collectors had healthcare backgrounds and undertook one-week training on the use of the study tools until they demonstrated competency in completeness and accuracy of test data entered. A 5% random selection of the case records was independently re-evaluated and cross-checked for accuracy and uniformity weekly during data collection. Agreement rates for data that were independently abstracted were consistently greater than 95%.

### Performance indicators

The primary effectiveness measures were 15 disease-specific process of care indicators. Performance indicators for this study were derived from the CPGs and ETAT+ programme after adaptation by the hospital staff in a consensus conference held in July 2008 using the modified nominal group technique [11]. The indicators spanned four domains in the continuum of care namely assessment, classification, treatment, and follow-up care for the initial 48 hours of admission. Composite indicators were developed to assess whether all individual tasks within the domain were performed (Table 1). The desired performance by the hospital staff for each of the individual indicators was set at 100% except for monitoring of intravenous fluid administration whose target was 50% in recognition of existing nurses' shortage.

**Table 1:** Definition of the composite indicators of processes of care for each diseases.

Domain of care	Criteria for considering the composite indicator achieved	Pneumonia	Dehydration	Severe malnutrition
**Assessment**	Patient adequately assessed if all the following signs are assessed	Level of consciousness ability to drink^a^, cyanosis, lower chest wall indrawing and respiratory rate	Level of consciousness, pulse character^b^, ability to drink^a^, sunken eyes and skin turgor (and duration of skin fold to return)	Oedema, and weight for height Z-score or visual assessment of degree of severe wasting
**Classification**	Consistent with CPGs/ETAT+ if any the corresponding terms are used	Very severe pneumonia, severe pneumonia,	Shock, severe dehydration, some dehydration and no dehydration	Severe malnutrition, oedematous malnutrition, protein energy malnutrition, marasmic kwashiorkor, kwashiorkor marasmus
**Treatment**	Consistent with CPGs if the following key treatment was prescribed at the correct dose and frequency (and duration for rehydration therapy)	Crystalline penicillin 50,000units/kg/dose X 4 per day (+/−20%) and/or Gentamicin 7.5 mg/kg/day X 1 per day (+/−20%)	Hartman's solution^c^ at 80–120 mls per kg if not given bolus for shock management or 56–120 mls per kg if given bolus for shock management given over 5–6 hours for patients ages 2–11 months and 2.5–3 hours in patients aged 12–59 months	100–130 mls/kg/day (+/−20%) of F75^c^
**Follow-up care**	Consistent with WHO/Kenya guidelines as adapted by the hospital staff	Evidence that doses of Crystalline penicillin were given as prescribed in the first 48 hrs of admission^d^	Evidence that intravenous fluid (IV) therapy for severe dehydration was monitored	Evidence that intake of feeds for severe malnutrition was monitored^e^.

a Patients documented to have altered consciousness were assumed that they are not able to drink if ability to drink is not documented while patients documented in the history as able to drink were assumed to have the sign ‘able to drink’.

b Patients documented as able to drink or alert were assumed not to have a weak pulse if pulse character was not documented.

c was either a manufactured product (depending on the availability) or milk-based solution prepared in the hospital that provided 75kcal and 0.9g of protein/100 ml.

d If dextrose added, correct if given at 2.4–6.0 mg/kg/min (approximates dextrose requirement for a sick child 3–5 mg/kg/min; +/−20%).

e Initial treatment is considered given on time if it is given within 12 hours of admission on the ward.

### Definitions

The definitions of the performance measures were based on KNH's adaptation of WHO/Kenya case management guidelines [9], [10], [13]. An indicator was considered achieved or correct if the care was consistent with CPGs, ETAT+ recommendations or staff consensus (Table 1). To define a population of critically sick children in the study we identified those who died within 5 days of admission. Critically sick patients were considered regularly reviewed by the clinicians if there was evidence from the medical notes that they were reviewed twice a day in the first 48 hours of admission. Likewise, they were regularly reviewed by the nurses if the temperature, respiratory rate and pulse rate were recorded every 6 hours for the first 48 hours of admission.

### Statistical analysis

All statistical analyses were carried out in STATA Version 11 (StataCorp, College Station, Texas). We present the results for each domain of care for each disease separately. However, as there were fewer critically sick patients, data for all diseases are combined for these analyses. Written consent was not obtained from the patients for their information to be stored in the hospital database and used for research as no such policy exists in the hospital. The data were thus analyzed anonymously.

#### Descriptive analysis

Patient characteristics are summarized as percentages with 95% confidence intervals (95% CI) or medians with inter-quartile range, as appropriate. Chi-square tests were used to compare categorical variables, and parametric (ANOVA) or nonparametric (Kruskal Wallis) tests were used to compare continuous variables with a normal or skewed distribution respectively.

#### Comparison of pre-intervention and post-intervention periods

We report performance of the composite indicators, dichotomised as achieved or not achieved, in pre-intervention (2005) and post-intervention (2009) periods with the primary effect being the absolute percentage change. We refer to an effect size of 50% or greater as very large, 20% to 49% as large, 10 to 19% as moderate, and below 10% as small based on previously used classifications [14], [15]. We did not make formal adjustment for multiple hypothesis testing. We therefore suggest interpretation of results from this study should consider the magnitude of the change and its temporal association with intervention rather than just the *P*-value.

#### Trend of change

We used chi-squared tests for trend to explore performance of the disease specific composite indicators across the four study periods. We excluded indicators for follow-up care of the critically sick children in this analysis because of small sample size.

### Ethics statement

Ethical approval was provided by the Kenyatta National Hospital/University of Nairobi Ethics and Research Committee (reference number KNH-ERC/01/480). Although case records from which data were abstracted had names, data collected were de-identified and unique study patient identifiers created. Further, this study was classified as audit and therefore informed consent from the participants was not found necessary by the institutional ethics reviews committee.

## Results

### Sample description

#### Case records

The retrieval rate of the sampled medical records was over 80% and there was no major variation across the study quarters for the three target diseases (data not shown). Of the medical records retrieved, 31% (1429/4577) of those with a discharge diagnosis of pneumonia, 51% (1536/3012) with dehydration, and 58% (1119/1922) with severe malnutrition were eligible for the inclusion. Common reasons for exclusion were presence of a co-morbidity that prompted treatment not consistent with the CPGs: i) For pneumonia cases: co-morbidities such as wheezy conditions, possible meningitis and severe malnutrition; ii) For dehydration cases: persistent diarrhoea or severe malnutrition and, iii) For severe malnutrition cases: known HIV infection or possible meningitis.

#### Severity of illness on admission

There was a substantial improvement in the use of CPG (and WHO) suggested syndromic classification of illnesses and in the documentation of key clinical signs during the study period (Table 2). This gives a misleading impression that there was an increase in serious forms of pneumonia and dehydration over the study period. This differential documentation of the severity of the target illnesses and key clinical signs over the study period compromises our ability to use documented severity of illness and to describe case-mix in exploratory analyses.

**Table 2:** Use of terms for severity of illness of the patients consistent with CPG/ETAT+ terms as recorded by clinicians on admission.

Target disease	Illness classification	2005 n (%)	2006 n (%)	2007 n (%)	2008 n (%)	2009 n (%)
**Pneumonia**						
	Very severe pneumonia	3/265 (1.1%)	4/283 (1.3%)	15/259 (5.8%)	26/312 (8.3%)	53/293 (18.1%)
	Severe pneumonia	40/265 (15.1%)	45/283 (15.0%)	92/259 (35.5%)	129/312 (41.4%)	166/293 (56.7%)
	Not classified a, b	222/265 (83.8%)	251/283 (83.7%)	152/259 (58.7%)	157/312 (50.3%)	74/293 (25.3%)
**Dehydration**						
	Shock	0/296 (0%)	5/308 (1.6%)	13/303 (4.3%)	8/334 (2.4%)	25/294 (8.5%)
	Severe dehydration	82/296 (27.6%)	80/308 (26.0%)	128/303 (42.2%)	120/334 (36.0%)	155/294 (52.7%)
	Some dehydration	139/296 (46.8%)	121/308 (39.3%)	116/303 (38.3%)	144/334 (43.4%)	79/294 (26.9%)
	No dehydration	1/296 (0.3%)	4/308 (1.3%)	9/303 (3.0%)	26/334 (7.8%)	7/294 (2.4%)
	Not classified a, c	75/296 (25.3%)	98/308 (31.8%)	37/303 (12.2%)	35/334 (10.5%)	28/294 (9.5%)
**Severe Malnutrition**						
	Severe malnutrition d	216/274 (78.8%)	172/223 (77.1%)	194/246 (78.9%)	135/179 (75.4%)	160/197 (81.2%)
	Not classified a	58/274 (21.2%)	51/223 (22.9%)	52/246 (21.1%)	44/179 (24.6%)	37/197 (18.8%)

a Not classified' means a diagnostic label captured by the target disease inclusion criteria but not consistent with ETAT+ terms was used.

b Includes pneumonia, bronchopneumonia and lobar pneumonia.

c Includes gastroenteritis and dehydration.

d Includes severe malnutrition, marasmus, marasmic kwashiorkor, kwashiorkor, oedematous malnutrition and protein energy malnutrition.

#### Patients' characteristics

There were more males than females with pneumonia in the pre-intervention period (59.6%) than in the post-intervention period (46.1%, p = 0.001). This pattern was consistent with that of the hospital data base of all pneumonia patients admitted in the corresponding periods. For dehydration, patients in the pre-intervention period were marginally younger (Kruskal-Wallis test, p = 0.003), of lower weight (Kruskal-Wallis test, p = 0.004) and had a longer duration of diarrhoea (Kruskal-Wallis test, p = 0.016) before seeking care in KNH than those in the post-intervention period. Patients with severe malnutrition in the pre-intervention period were older (p = 0.015) and marginally heavier (Kruskal-Wallis test, p = 0.056) than those in the post-intervention period (Table 3). This variation over time in patients' age and sex observed in our data (and wider hospital data) remains difficult to explain, especially as high quality health service data from other hospitals are not available for comparison.

**Table 3:** Patients' characteristics (2005–2009) and comparison of characteristics between the pre-intervention (2005) and post-intervention periods (2009).

Target disease	Patients characteristics	2005	2006	2007	2008	2009	*P*-value 2005 vs. 2009
**Pneumonia**							
	Male n (% )	158/265 (59.6%)	174/300 (58.0%)	144/259 (55.6%)	172/312 (55.1%)	135/293 (46.1%)	0.001^a^
	Median (IQR)age in months	8 (4–14)	8(5–14)	8 (4–14)^c^	8 (5–15)	7 (4–14)	0.89^b^
	Median(IQR weight)in kilograms	7.0 (5.5–9.0)^c^	7.1 (5.7–9.0) d	7.2 (6.1–8.7)	7.1^d^ (5.8–9.1)	7.0 (5.7–9.0)	0.69^b^
	Median (IQR) duration of cough in days	4 (3–7)	4 (3–7)	4 (3–7)	4(3–7)	4 (3–7)	0.28^b^
**Dehydration**							
	Male n (% )	156/297 (52.5%)	182/308 (59.1%)	165/303 (54.5%)	194/334 (58.1%)	139/294 (47.3%)	0.200^a^
	Median age in months (IQR)	7(5–11)	8 (5–12)	9(5–14)	8(6–12)	9(6–12)	0.003^b^
	Median weight in kilogram (IQR)^d^	7.0 (5.9–8.1)	7.2 (6.0–8.4)	7.2 (6.2–8.5)^c^	7.3 (6.1–8.5)^c^	7.4 (6.3–8.6)^d^	0.004^b^
	Median duration (IQR) of diarrhoea in days	4(3–6)	3 (2–5)	3 (2.5–5)	3(3–5)	3 (2–5)	0.016^b^
**Severe malnutrition**							
	Male n (% )	130/274 (47.5%)	117/223 (52.5%)	124/246 (50.4%)	79/179 (44.1%)	82/197 (41.6%)	0.21 a
	Median (IQR) age in months	14 (10–22)	12 (9–18)	12 (9–17)	14 (9–20)	13 (9–18)	0.015^b^
	Median (IQR) weight in kilogram^d^	6.5 (5.5–8.0)^c^	6.0 (5.2–7.0)	6.0 (5.1–7.0)^c^	6.0 (5.2–7.3)	6.3 (5.3–7.3)	0.056^b^

a Chi square test.

b Kruskal Wallis test.

c Denominator does not include three patients with missing values.

d Denominator does not include one patient with missing values.

### Effects of the intervention

#### Performance of assessment and classification indicators

The intervention produced large improvement of the composite indicators for assessment of pneumonia (+27.1%, 95% CI: +21.7 to +32.6%) and dehydration (+23.5%, 95% CI: +18.6 to +28.3%). There was also a large improvement in classification of severity of pneumonia syndromes (+58.5%, 95% CI: +51.9 to +65.2) and a modest improvement for dehydration (+15.7%, 95% CI: +9.8 to +21.7). Nevertheless, the signs used in classification were not always consistent with CPGs recommendation. For example, 18/40 (45.0%) and 35/166 (21.1%) of patients classified as severe pneumonia in pre-intervention period and post-intervention periods respectively had one or more features of very severe pneumonia (altered consciousness, inability to drink or cyanosis). The intervention did not result in significant changes in the assessment or classification of patients with severe malnutrition (Table 4).

**Table 4:** Effects of the intervention on adequacy of assessment and illness classification.

Domain of care	Composite and individual indicators	Patients who achieved the indicator in the pre-intervention period n (%,95% CI)	Patients who achieved the indicator in the post-intervention period n (%,95% CI)	Effect size – absolute difference in percentage %; (95% CI)	*P*-value
**Assessment of pneumonia patients**					
	**Composite indicator**	**5/265 (1.9%; 0.6–4.3)**	**85/293 (29.0%; 23.9–34.6)**	**+27.1%; (+21.7 to +32.6)**	**<0.001**
	Level of consciousness	58/265 (21.9%; 17.1–27.4)	218/293 (74.4%; 69.0–79.3)		
	Ability to drink	100/265 (37.7%; 31.9–43.9)	155/293 (52.9; 47.0–58.7)		
	Cyanosis	164/265 (61.9%; 55.7–67.8)	220/293 (75.1%; 69.7–79.9)		
	Lower chest wall indrawing	49/265 (18.5%; 14.0–23.7)	238/293 (81.2%; 76.3–85.5)		
	Respiratory rate	206/265 (77.7%; 72.2–82.6)	255/293 (87.0%; 82.6–90.7)		
**Assessment of dehydrated patients**					
	**Composite indicator**	**0/297 (0%; N/A)**	**69/294 (23.5%; 18.7**–**28.7)**	**+23.5%; (+18.6 to 8.3)**	**<0.001**
	Level of consciousness	169/297 (56.9%; 51.1–62.6)	247/294 (84.0%; 79.3–88.0)		
	Ability to drink^b^	88/297 (29.6%; 24.5–35.2)	136/294 (46.3%; 40.5–52.1)		
	Pulse character	74/297 (24.9%; 20.1–30.2)	250/294 (85.0%; 80.4–88.9)		
	Sunken eyes	86/297 (29.0%; 23.8–34.5)	206/294 (70.1%; 64.5–75.2)		
	Skin turgor	19/297 (6.4%; 3.9–9.8)	211/294 (71.8%; 66.3–76.8)		
**Assessment of severe malnutrition**					
	**Composite indicator**	**120/274 (43.8%; 37.8**–**49.9%)**	**78/197 (39.6%; 32.7**–**46.8)**	**−4.2; (−13.2 to +4.8)**	**0.36**
	Oedema	193/274 (70.4%; 64.7–75.8)	126/197 (64.0%; 56.8–70.7)		
	Visible severe wasting	161/274 (58.8%; 52.7–64.7)	103/197 (52.3%; 45.1–59.4)		
	Height	0/274 (0%; N/A)	51/197 (25.9%; 19.9–23.6)		
**Classification of illness consistent with the CPGs**					
	**Pneumonia**	**43/265 (16.2%; 12.0**–**21.2)**	**219/293 (74.7%; 69.4**–**79.6)**	**+58.5%; (+51.9 to+65.2)**	**<0.001**
	**Dehydration**	**222/297 (74.8%; 69.4**–**79.6)**	**266/294 (90.5%; 86.5**–**93.6)**	**+15.7%; (+9.8 to +21.7)**	**<0.001**
	**Severe malnutrition**	**216/274 (78.8%; 73.5**–**83.5)**	**160/197 (81.2%; 75.1**–**86.4)**	**+2.4%; (−0.49 to +9.6]**	**0.52**

#### Performance of treatment indicators

Among children with pneumonia, prescription of correct dosages of crystalline penicillin improved from 51.7% in 2005 to 90% in 2009 (Table 5). This large increase of +38.2% (95% CI: +31.2 to +45.2) was mainly attributed to reduced under-dosing from 39.2% (95% CI: 33.3–45.4) to 2.1% (95% CI: 0.7–4.4). The correct prescription of gentamicin for pneumonia was observed in 19.9% (95% CI: 14.5–26.3) of children in 2005, and 88% in 2009, showing an absolute improvement of +68.0% (95% CI: +60.1 to +75.8).

**Table 5:** Effects of intervention on selected treatment practices^a^.

Composite and corresponding individual tasks indicators		Patients whose treatment was consistent with CPGs in the pre-intervention period n(%; 95% CI)	Patients whose treatment was consistent with CPGs in the post-intervention period n (%; 95% CI)	Effect size – difference in percentage % (95% CI)	*P*-value^a^
**Antibiotics therapy for Pneumonia**	**Dose and frequency of crystalline penicillin consistent with CPGs** b	**137/265 (51.7%; 45.5**–**57.9)**	**258/287 (89.9%; 85.8**–**93.1)**	**+38.2%; (+31.2 to +45.2)**	**<0.001**
	Patients who received less that 80% of the recommended dose of crystalline penicillin	104/265 (39.2%, 33.3–45.4)	6/287 (2.1%; 0.7–4.4)		
	**Dose and frequency of gentamicin consistent with guidelines** c	**38/191 (19.9%; 14.5**–**26.3)**	**123/140 (87.9%; 81.3**–**92.8)**	**+68.0%; (+60.1 to +75.8)**	**<0.001**
	Frequency of gentamicin consistent with ETAT+ guidelines	46/191 (24.1%; 18.2–30.8)	138/140 (98.6%; 94.9–99.8)		
**Fluid therapyfor dehydration**	**Intravenous fluid therapy consistent with ETAT+ guidelines** d, e	**100**/265 **(37.7%; 31.9**–**43.9)**	**177/286 (61.8%; 60.0**–**67.5)**	**+24.2% (+16.0 to +32.3)**	**<0.001**
	Type of IVF consistent with CPGs	219/265 (82.6%; 77.5–87.0)	251/286 (87.8%; 83.4–91.3)		
	Volume of IVF consistent with CPGs	160/265 (60.4%;54.2–66.3)	205/286 (71.7%; 66.1–76.8)		
	Duration of IVF consistent with CPGs	147/265 (55.5%; 49.3–61.6%)	213/286 (74.5%; 69.0–79.4)		
**Feeds for severe malnutrition**	**Feed prescription consistent with CPGs** f	**25/274 (9.1%; 6.0**–**13.2)**	**135/197 (68.5%; 61.5**–**74.9)**	**+59.5%; (+52.1 to +66.7)**	**<0.001**
	Patients with feeds prescribed	233/274 (85.0%; 80.3–89.0)	176/197(89.3%; 84.2–93.3)		
	Type of feed consistent with CPGs	31/233 (13.3%; 9.2–18.4)	142/176 (80.7%; 74.1–86.2)		
	Volume of consistent with CPGs	124/233 (53.2%; 47.0–60.2)	158/176 (89.8%; 84.3–93.8)		

a Chi square test.

b Composite indicator; achieved if dose and frequency of crystalline penicillin is consistent with CPGs.

c Composite indicator; achieved if dose and frequency of gentamicin is consistent with CPGs.

d Composite indicator; achieved if type, volume and duration of IVF is consistent with CPGs.

e 22 records for 2005 and 3 for 2009 were excluded in the analysis for treatment due to serum sodium >145 or <135 mmol/l. In addition 11 and 5 records in 2005 and 2009 respectively were excluded because there was no evidence that IVF was continued on the ward and diagnosis of either some dehydration or no dehydration was made.

f Composite indicator; achieved if type and volume of feeds for severe malnutrition is consistent with CPGs.

Correct intravenous fluid therapy for dehydrated patients was prescribed for 37.7% of children in 2005 and 61.8% of children in 2009 giving an effect size of +24.2% (95% CI: +16.0 to 32.3). Majority of patients (>85%) with severe malnutrition had feeds prescribed on admission in both the pre-intervention and post-intervention periods. However, the intervention resulted in a very large improvement (+59.5%, 95% CI: +52.1 to +66.7) in the correctness of these prescriptions (Table 5).

Despite improvement in prescription practices, documentation that prescribed treatment was administered in the first 48 hours of admission remained poor in both pre-intervention and post intervention periods (Table 6).

**Table 6:** Effect of intervention on the administration of the prescribed treatment in the first 48 hours of admission.

Task in administration of treatment	Patients who had the task achieved in pre-intervention period n (%; 95% CI)	Patients who had the task achieved in post-intervention period. n (%; 95% CI)	effect size – difference in percentage % (95% CI)	*P*-value
Evidence that 8 doses crystalline was given for children with pneumonia a	20/154 (13.0%; 7.6–18.4)	31 (16.6%; 11.2–22.0)	+3.6% (−3.9 to +11.1)	0.36 b
Median doses of crystalline penicillin (IQR) given for children with pneumonia c	5.5 (4.5–7.0)	6.5 (5.5–7.5)	-	0.54 c
Intravenous fluid therapy for severe dehydration monitored d	2/285 (0.7%; 0.9–2.5)	16/285 (5.6%; 3.2–9.0)	+4.9% (+2.1 to +7.8)	0.001 b
Patients with severe malnutrition that had feeds prescribed and whose feeds intake was documented in the feed chart e	3/223 (1.3%; −0.2 to 2.9)	13/170 (7.6%; 3.6–11.7)	+6.3% (+2.0 to +10.6)	0.002^b^

a Analysis restricted to patients alive after 2 days, crystalline penicillin prescribed as four times a day and not stopped during the first 2 days of admission.

b Chi square test.

c Kruskal Wallis test.

d Analysis restricted to patients with evidence that intravenous fluid (IVF) was prescribed on the ward and were alive after one day of admission.

e Analysis restricted to patients who had feed prescribed and were alive after 1 day of admission.

#### Review of critically sick patients

Eleven per cent (93/836) of all study patients in the pre-intervention period and 6% (45/784) of patients in the post-intervention period were critically sick. Documented review of the patients by both nurses and clinicians in the first 48hrs of admission was very poor in both pre-intervention and post-intervention periods (Table 7).

**Table 7:** Effect of intervention on review of patients who died in the first 48 hours of admission.

Composite indicator	Individual task	Patients who had the task/indicator achieved in the pre-intervention period n (%; 95% CI)	Patients who had the task/indicator achieved in the post-intervention period n (%; 95% CI)	effect size – difference in percentage % (95% CI)	*P-*value^c^
Nurses' review and documentation of vital signs^a^	6houly review of vital signs b	1/93 (1.1%; 0.0 to −5.8)	3/45 (6.7%; 1.4–18.2)	+5.6% (−2.0 to +13.2)	0.066
	Temperature	1/93 (1.1%; 0.0 to −5.8)	3/45 (6.7%; 1.4–18.2)		
	Respiratory rate	1/93 (1.1%; 0.0 to −5.8)	3/45 (6.7%; 1.4–18.2)		
	Pulse rate	1/93 (1.1%; 0.0 to −5.8)	3/45 (6.7%; 1.4–18.2)		
Clinicians' review a	Review of the critically sick patients 12hrly	4/93 (4.3%; 1.2–10.6)	2/45 (4.4%; 0.5–15.1)	0.1% (−7.2 to +7.4)	0.96
	Clinicians review 6hrs before death	21/93 (22.6%; 14.6–32.4)	17/45 (37.8%; 23.8–53.5)		

a Analysis restricted to patients who died within 5 days of admission but were alive on the first day of admission.

b All the three vital signs (temperature, pulse and respiratory rate) assessed.

c Chi square test.

#### Case fatality rates

Though this study was not designed to evaluate change in mortality rate, the mortality rate for children admitted with pneumonia dropped from 15.1% (40/265) in the pre-intervention period to 6.5% (19/293) in the post-intervention period. Mortality for children admitted with dehydration dropped from 17.9% (53/297) to 8.8% (26/294). Mortality rate for severe malnutrition dropped from 29.9% (82/284) to 22.3% (44/197). However, as explained above, inadequate case data precluded attempts to explore whether changing case-severity contributed to these reductions. Overall, deaths amongst children admitted with the target diseases that occurred in the first 48 hours of admission accounted for 45.1% (79/175) 49.4% (44/89) deaths in the pre-intervention and post-intervention periods respectively.

#### Trend of change

There was consistent improvement of performance for 10/13 of the composite indicators assessed over the four sequential periods (Table 8). Results suggest initial improvement in prescription of gentamicin, crystalline penicillin and feeds for malnourished patients during piloting of the CPGs and ETAT+ training (period 1). For seven of the 13 indicators, improvement began in the period of initial scaling up of CPGs and training (period 2) and for five indicators, there was some suggestion of further improvement during the period of reinforcement (period 3). Three indicators (administration of penicillin, and assessment and classification of severe malnutrition) did not show significant variation during any of the four study periods (Table 8).

**Table 8:** Test for trend of performance of the composite indicators for each target disease across the periods.

Target disease	Composite indicator	Period 0 Proportion (%, 95% CI)	Period 1 Proportion (%, 95% CI)	Period 2 Proportion (%, 95% CI)	Period 3 Proportion (%, 95% CI)	P-value of chi squared for trend
**Pneumonia**						
	Adequately assessed	5/265 (1.9%; 0.6–4.3%)	5/300 (1.7%; 0.5–3.8%)	62/403 (15.4%;12.0–19.2%)	107/461 (23.2%;19.4–27.3%)	<0.001
	correctly classified	43/265 (16.2%; 12.0–21.2%)	49/300 (16.3%;12.3–21.0)	178/403 (44.2%; 39.3–49.1%)	303/461 (65.7%; 61.2–70.1%)	<0.001
	Dose of crystalline penicillin consistent with CPGs	137/265 (51.7%; 45.5–57.9%)	209/300 (69.7%; 64.1–74.8%)	343/399 (86.0%; 82.2–89.2)	409/452 (90.5%; 87.4–93.0%)	<0.001
	Dose of gentamicin consistent with CPGs	38/191(19.9%;14.5–26.3%)	167/243(68.7%; 62.5–74.5%)	175/242 (72.3%; 66.2–77.9%)	221/244 (90.6%; 86.2–93.9%)	<0.001
	Administration of crystalline penicillin	20/154 (13.0%; 8.1–19.3%)	20/163 (12.3%; 7.7–18.3%)	19/216 (8.8%; 5.4–13.4%)	43/275 (15.6%; 11.6–20.5%)	0.463
**Dehydration**						
	Adequately assessed	0/297	1/308 (0.3%; 0.0–1.8%)	45/490 (9.2%; 6.8–12.1%)	108/441 (24.5%;20.5–28.9%	<0.001
	correctly classified	222/297 (74.5%; 69.4–79.6%)	210/308 (68.2%; 62.7–73.3%)	429/490 (87.5%; 84.3–90.3%)	402/441 (91.2; 88.1–93.6%)	<0.001
	IVF therapy consistent with CPGs	100/265 (37.5%; 31.9–43.9%)	118/290 (40.7%; 35.0–46.6%)	249/473 (52.6%; 48.0–57.2%)	260/422 (61.2%; 56.8–66.3%)	<0.001
	IVF monitored	2/297 (0.1%; 0.1–2.4%)	1/308 (0.3%; 0.0–1.8%)	7/490 (1.4%; 0.58–2.92%)	18/441 (4.1%; 2.4–6.4%)	<0.001
**Severe malnutrition**						
	Adequately assessed	120/274 (43.8; 37.8–49.9%)	93/223 (41.7; 39.2–48.5%)	125/335 (37.3; 32.1–42.7%)	114/287 (39.7; 34.0–45.6%)	0.20
	correctly classified	216/274 (78.8; 73.5–83.5%)	172/223 (77.1; 71.1–82.5%)	264/335 (78.8%; 74.0–83.1%)	225/287 (78.4%; 73.2–83.0%)	0.98
	Feeds therapy consistent with CPGs	25/274 (9.1; 0.6–13.2%)	108/223 (48.4; 41.7–55.2%)	185/335 (55.2; 49.7–60.6%)	209/287 (72.8; 67.2–77.9%)	<0.001
	Feeds monitored	3/223 (1.3; 0.2–3.9%)	1/198 (0.5%; 0–2.7%)	13/271 (4.8%; 2.6–8.1%)	20/247 (8.1%; 5.0–12.2%)	<0.001

## Discussion

Before CPGs/ETAT+ training, care for common serious childhood illnesses in KNH, a university teaching hospital, was largely inconsistent with best-practice recommendations as defined by WHO and endorsed within Kenya. Only 2/15 indicators had baseline performance of over 70%, while 8/15 indicators had performance below 10%, suggesting little impact of any previous efforts to disseminate WHO and local guidelines. This poor uptake of international recommendations for care is consistent with findings in district hospitals in this country [3], [16], [17], earlier studies in KNH [18] and other studies in low-income countries [1], [5]. Our data however indicate that 5/15 indicators achieved performance of over 70% in the post-intervention period which we consider acceptable performance of the composite indicators in this study. We observed an absolute increase in performance of over 20% for 7/15 key indicators, with three improving by over 50% (pneumonia classification, gentamicin dosage and prescription of feeds for the malnourished). All quality-of-care indicators for pneumonia and diarrhoea patients that relied on individual clinicians' adherence showed significant improvement. Yet all the indicators whose achievement was based on more sustained team efforts (evidence of administration of prescribed treatment and regular review of the critically sick patients over 24 to 48 hours) showed no significant improvement. These findings suggest that improvements in areas that require teamwork are less easy to achieve with primarily educational interventions targeting individuals. This is perhaps not surprising as teamwork is socially constructed and shaped by organizational and professional norms and context [2], [19]. In other settings, poor teamwork has resulted from a fragmented division of labour linked to power struggles associated with professional boundaries and hierarchies [19].

Examination of the individual tasks aggregated into composite indicators (data not shown) allowed us to identify suboptimal health workers' practices and gave us insight into specific clinical decision-making processes. There was generally marked improvement in patient assessment. However, for assessment of pneumonia and diarrhoea, the sign ‘ability to drink’ achieved the lowest performance. This sign has not been emphasised in mainstream academic paediatric teaching, despite it being a predictor of death in seriously ill children [20]. We speculate that trainee paediatricians consider it a sign for less well trained health-workers. There were also suggestions from the data that improved use of recommended terms for severity classification was not necessarily associated with better documentation and use of the key physical signs on which severity classification is based. This suggests that clinicians may be assigning classifications intuitively instead of carefully using recommended algorithms.

There was some evidence of improvements in recommended processes of care increasing with intensity (dose) of the interventions. However, the adoption rate of the best practices was variable across diseases and the study periods. Improvement in prescription practices began during piloting of the intervention. This was perhaps related to the attendance at a guidelines development workshop by some paediatric trainees where drug dose guidelines were discussed and endorsed followed by rapid informal dissemination of knowledge and CPGs amongst this clinical group [8]. In addition, staff were perhaps familiar with drug dosage guidelines as KNH had produced these for many years. Changes of prescription behaviour might therefore represent less of a change and be more likely to be consistent with norms promoting correct prescribing. However, changes in prescription of rehydration therapy, perhaps more complex than the other treatments, were observed only after formal dissemination of the guidelines.

Contrary to our expectation, the composite indicator for severe malnutrition assessment, comprising only two individual tasks which we considered to be simple was not adopted and had poorer performance than those for assessment of diarrhoea and pneumonia which spanned five tasks each. This appears to defy the simple premise that the more steps in a pathway, the less likely there is to be success [21]. There were also areas in which the intervention had no significant effect despite there being very poor performance at baseline, though studies elsewhere suggest that educational interventions are more effective in the context of low baseline adherence to guidelines [22]. Taken together our results confirm that even within a single setting the adoption rate of best-practices is unpredictable [23], [24]. These results are consistent with debates drawing on complexity and system theories [25], [26] and underline the value of concurrent qualitative enquiry to understand such variation.

It is hard to interpret the apparently large falls in case fatality rates especially as these cannot be appropriately adjusted for disease severity and there are no comparable control site data. Further, while we demonstrated improvements in domains of care studied independently the effect on patients' outcomes will likely reflect the way multiple domains of care are improved within one patient journey, something our data do not necessarily capture. Despite this our observations do raise the possibility that adherence to simple and low-cost recommendations, as stipulated in WHO case management guidelines, combined with perhaps other improvements in service provision, may reduce case fatality of common serious illnesses in tertiary care facilities, a finding consistent with other studies [27]. Formal investigation of possible effects on mortality may therefore be warranted although this would require appropriately designed, comparative studies that would be challenging to undertake.

Despite some improvements observed in this study, the overall frequency of errors in the management of common serious illnesses at a university teaching hospital expected to train manpower that is responsive to societal needs remains of concern. In particular, it should challenge academic staff and national professional bodies concerned with establishing and maintaining standards of expertise [28] to continue efforts to improve further quality of care.

Poor documentation of the care provided by the health professionals may explain, in part, the observed errors in the management. Simple approaches to improve documentation of illness that might support introduction and use of guidelines could include use of critical care pathways or standardised record forms that have proven valuable in other settings [4], [16]. However, health systems are complex and adoption of best-practices may be determined by multiple causes that may interact in a non-additive fashion. There is clearly a need to understand what constrains better practice and uptake of guidelines. In work to be reported elsewhere, we will attempt to explain the observations reported here in considerably greater detail. Preliminary observations however suggest that there are major challenges in providing an integrated and coordinated approach to leadership and management in what is a complex environment with multiple professions responsible for care. Further challenges include inadequate attention paid to supervision and erosion of professional values linked, perhaps, to poor staff morale.

### 

#### Limitations

The study had a number of limitations. First, we acknowledge that a before and after design without control data makes it difficult to infer causal relationships between the intervention and the outcomes [29]. Exploration of dose-effect relationships as we report does, however, provide some ability to strengthen any inferences. Deeper understanding of how and why effects emerged can also be provided by detailed qualitative work, contributing insights that are not gained from an epidemiological design [30], [31], [32], [33], [34]. We will report separately on data collected during the 18 months of reinforcement of CPG uptake to further explain the results we have reported here.

Secondly, when basing work on review of records we assume implicitly that the written records reflect actual practice, a potential limitation. This analysis, however, is based on indicators of practices that hospital staff felt should be documented to avoid compromising the ability of the clinical team to provide effective treatment and follow-up for patients. Further, several indicators were based on treatment prescriptions that were routinely well documented throughout the entire study period. Such prescriptions arguably provide the most robust link between a clinician's intentions and the treatment actually received.

Finally, the intervention used assumed the knowledge embodied in the CPGs is uncontested and that rationally, increased adherence to the guidelines merely requires an increase in dissemination. This model ignores the hidden or poorly illuminated values or needs that influence practitioner and organizational behaviour.

#### Conclusions

There are very few published studies evaluating the impact of dissemination of guidelines for multiple serious childhood illnesses in a large teaching hospital in a LIC. Our data complement that at outpatient level [35] and from district hospitals, [1], [3], [4], [5], [16], [17] tending to show a consistent pattern of relatively poor quality care for common, serious diseases in the absence of active efforts to implement recommended practices [4], [16], [17]. Our results demonstrate that active dissemination of a combination of CPGs and ETAT+ training, can result in considerable improvements in adherence to national and international recommendations, impacting on the quality of in-patient care, particularly for tasks that rely on competence of individual clinicians. These findings are consistent with those of a recent cluster randomised trial in Kenya [16]. However, our experience in this study, and published reports [36] suggest that considerably more attention must be given to broader implementation strategies that target institutional and organisational aspects of service delivery to improve team-based quality of care.
